# Identification of smile events using automated facial expression recognition during the Autism Diagnostic Observation Schedule (ADOS-2): a proof-of-principle study

**DOI:** 10.3389/fpsyt.2025.1497583

**Published:** 2025-05-01

**Authors:** Maria Dotzer, Ulrike Kachel, Jan Huhsmann, Hendrik Huscher, Nils Raveling, Klaus Kugelmann, Stefanie Blank, Isabel Neitzel, Michael Buschermöhle, Georg G. von Polier, Daniel Radeloff

**Affiliations:** ^1^ Department of Child and Adolescent Psychiatry, Psychotherapy and Psychosomatics, University Hospital Leipzig, Leipzig, Germany; ^2^ KIZMO GmbH - Clinical Innovation Center for Medical Technology, Oldenburg, Germany; ^3^ SpeechCare GmbH, Leverkusen, Germany; ^4^ Branch for Hearing, Speech and Audio Technology HSA, Fraunhofer Institute for Digital Media Technology IDMT, Oldenburg, Germany; ^5^ Research Unit of Language and Communication, Department of Rehabilitation Sciences, TU Dortmund University, Dortmund, Germany

**Keywords:** facial expression recognition, ADOS, autism diagnosis, digital diagnosis, ROC, smile recognition, diagnosis software, early autism diagnosis

## Abstract

**Introduction:**

The diagnosis of autism spectrum disorder (ASD) is resource-intensive and associated with long waiting times. Digital screenings using facial expression recognition (FER) are a promising approach to accelerate the diagnostic process while increasing its sensitivity and specificity. The aim of this study is to examine whether the identification of smile events using FER in an autism diagnosis utilisation population is reliable.

**Methods:**

From video recordings of children undergoing the Autism Diagnostic Observation Schedule (ADOS-2) due to suspected ASD, sequences showing smile and non-smile events were identified. It is being investigated whether the FER reliably recognizes smile events and corresponds to a human rating.

**Results:**

The FER based on the facial action unit mouthSmile accurately identifies smile events with a sensitivity of 96.43% and a specificity of 96.08%. A very high agreement with human raters (κ = 0.918) was achieved.

**Discussion:**

This study demonstrates that smile events can in principle be identified using FER in a clinical utilisation population of children with suspected autism. Further studies are required to generalise the results.

## Introduction

Over the past two decades, considerable technical progress has been made in the automated analysis of facial expressions, language and gestures. Data processing tools are increasingly available on mobile devices and are therefore accessible to a wide range of users, opening up a variety of potential applications, for example in healthcare diagnostics and rehabilitation (1–4).

Autism spectrum disorder (ASD) is characterized by abnormalities in social communication (including reduced social initiative, social smile and eye contact, reduced gestures, reduced mimic), in speech and language and movement stereotypes (5). Currently, the diagnosis of ASD relies on the assessment of experts, with screening-instruments, interviews and structured behavioral observations, such as the Autism Diagnostic Observation Schedule (ADOS-2) (6, 7). The high demand for an ASD diagnostic, the low specificity of the screening systems (5) and the low relative availability of diagnostic resources result in long waiting times and late diagnosis internationally, which some authors refer to as a waiting list crisis (8). Automating parts of the diagnostic process through facial expression recognition (FER) could provide an objective, accessible, and resource-efficient screening tool, particularly in structurally weak regions (9).

This study is part of the IDEAS project (*Identification of autism using* sp*eech and facial expression analysis)* which examines the technical feasibility and clinical potential of automated analysis methods for ASD screening (10). It focuses on the identification of smile events, as social smiles play a crucial role in ASD diagnostics (5). Research indicates, that the non-verbal communication skills of autistic children differ from that of healthy controls, for instance through reduced social smiling, pointing gestures and eye contact (5, 11, 12). Therefore, one of the key points of the clinician’s evaluation during ADOS assessments, is to observe whether a child initiates, shares or reciprocates smiles, a fundamental skill for social interaction (5). This proof-of-principle study aims to determine whether smile events of an individual can be reliably automatically detected with FER.

Most facial expression analysis software [for an overview, see (12)] include an option for identifying facial Action Units (AU), a concept based on the Facial Action Coding System (FACS) developed by Ekman (13). The identification of smile events is based on AU 6 (cheek raiser) and AU 12 (lip corner puller), which are associated with the expression of joy (14, 15). While previous studies have demonstrated high accuracy in identifying smile events in controlled environments in healthy adults (14, 15), its application in naturalistic diagnostic settings presents challenges. These include variability in recording conditions, occlusions (e.g., hands or objects covering the face), movement artifacts, and the spontaneous nature of expressions, all of which can affect detection accuracy (15). It is therefore necessary to test the reliability of the FER in a challenging population, namely freely moving children with autistic symptoms.

In the current study, we address the following hypothesis: automated FER (based on AU 6 and AU 12 events) identifies smile events in video sequences of ADOS videos with high sensitivity/specificity and thus shows high agreement with human ratings.

## Methods

### Study participants and psychological assessment

Nine children (age: *M* = 10.24; *SD* = 3.84; range: 5.63-16.78; sex: 2 female, 7 male) were recruited via the outpatient ASD unit of the Department of Child and Adolescent Psychiatry, Psychotherapy and Psychosomatics, University Hospital Leipzig. All participants were presented at the clinic due to indications of ASD and were undergoing diagnostic clarification. All participants exhibited fluent speech.

The participants underwent standardized ASD diagnostics, with the results taken from the medical records. The ASD screening instrument Social Communication Questionnaire (SCQ; German version) (13) was completed for each participant. SCQ raw values ranged from 6 to 27 (*M* = 16.89; *SD* = 7.64). The ADOS-2 (German version) (6) was performed by an experienced certified examiner. One participant was assessed with ADOS module 2 (total score 6), 7 participants with module 3 (total score *M* = 6.14; *SD* = 2.91; range: 3-12), and one participant with module 4 (total score 5). This indicates that the autistic symptoms observed in this sample were of a moderate severity. The subject’s IQ ranged from 81 to 122 (*M* = 103.8; *SD* = 12.25). A single participant was diagnosed with ASD following the conclusion of the diagnostic process.

### Video recording and processing

**Figure 1:**
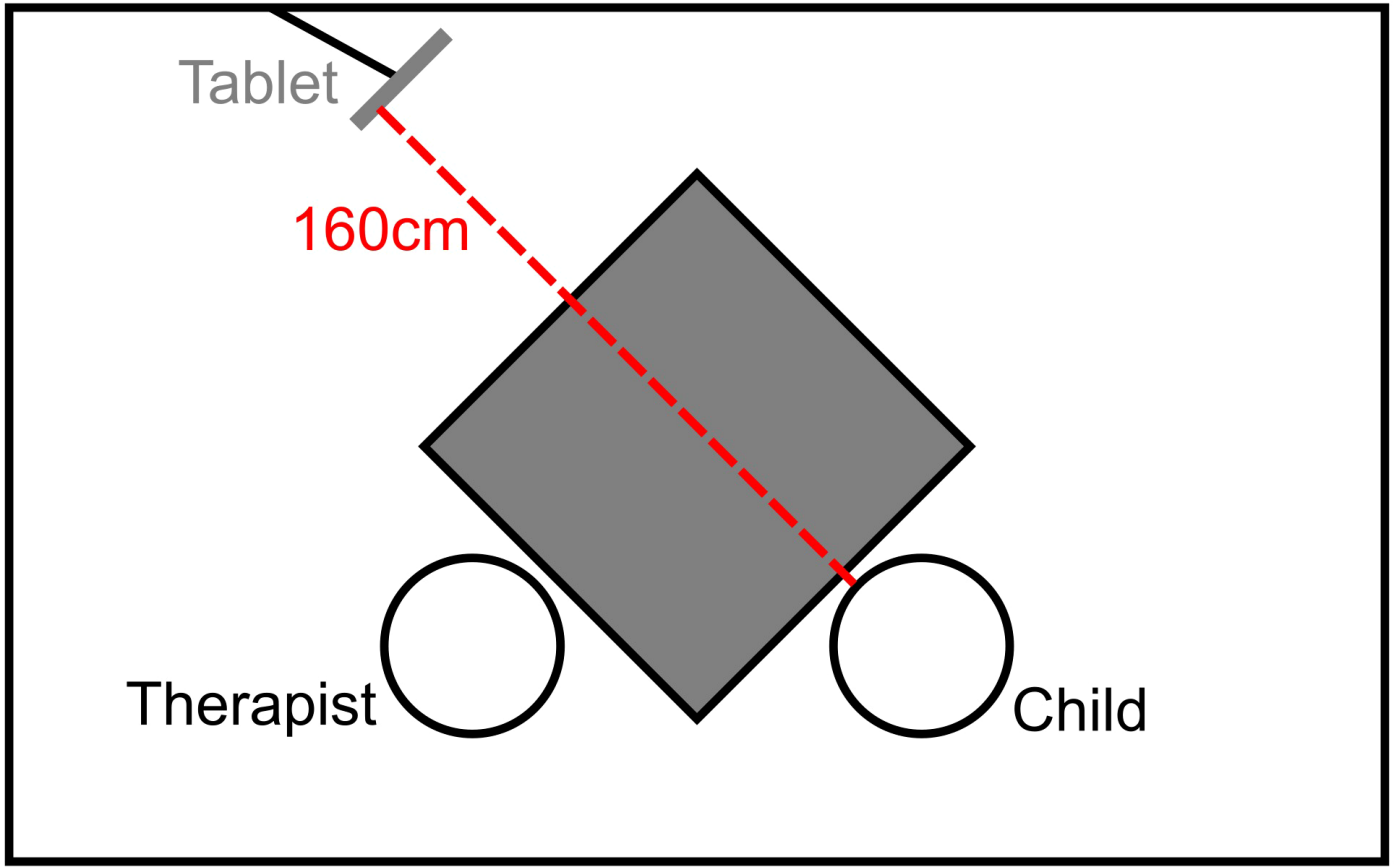
Schematic illustration of the recording setting. The therapist conducting the ADOS and the child were sitting across the corner of a table. The tablet was oriented towards the child’s face, and parallel to the edge of the table.

The children were video recorded during ADOS sessions. The video was recorded using the front camera of an Apple iPad Pro (4^th^ generation, iOS 17.3.1), wall-mounted at eye level in a distance of around 160 cm to the child’s face (see **Figure 1**). The tablet screen was covered to ensure minimal distraction.

**Figure 2:**
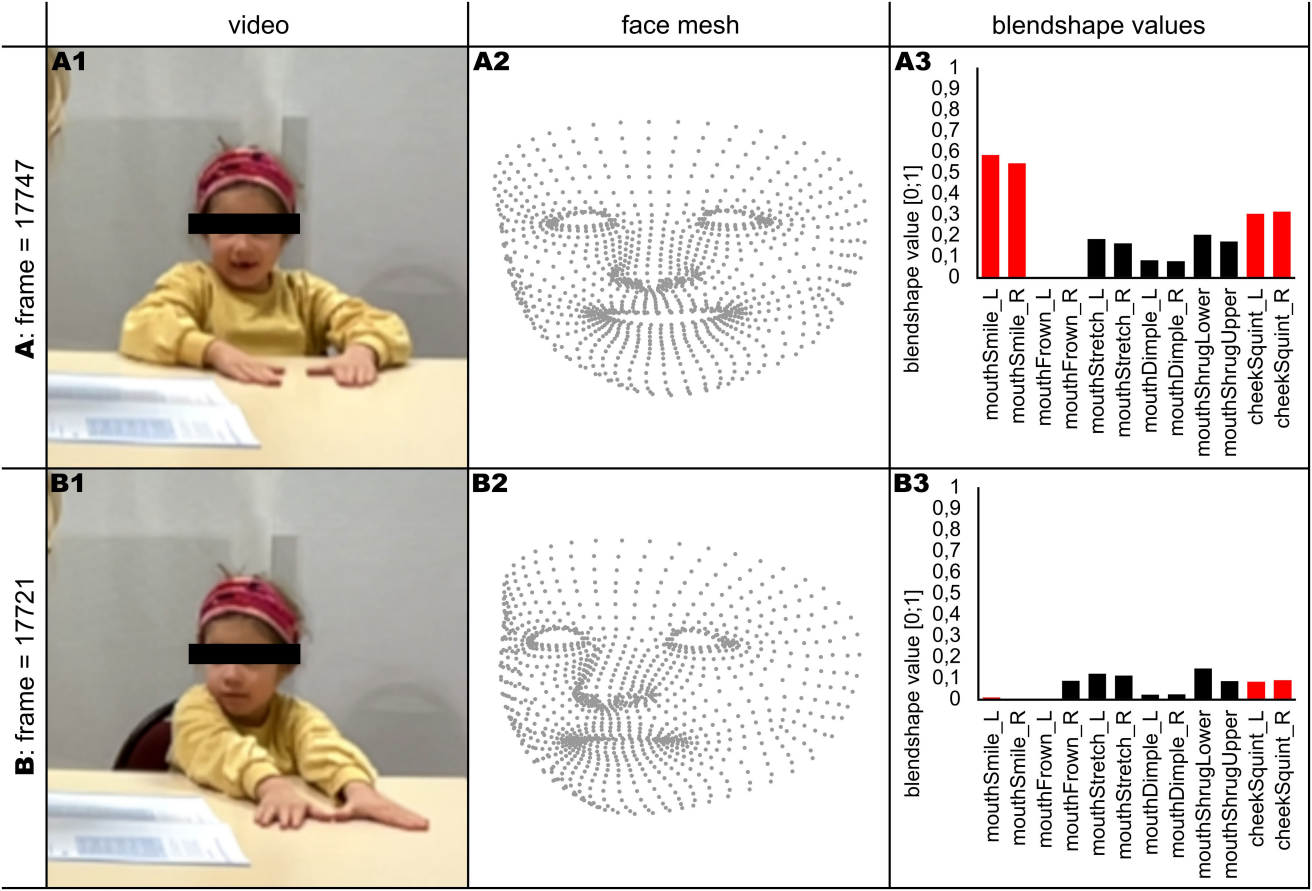
Data of two example frames **(A, B)** A1/B2 show the video image of the respective frame. A2/B2 show the mesh of the recorded face coordinates, from which blendshape values are derived. A3/B3 show a selection of blendshape values for the respective frame. Higher values indicate a more pronounced expression, lower values indicate a more neutral expression. The blendshapes of interest (mouthSmile_L, mouthSmile_R, cheekSquint_L and cheekSquint_R) are highlighted in red.

A FER software developed within the IDEAS project was applied for data acquisition. The software is based on the Apple ARKit, which enables real-time face tracking and identification of facial features in form of a 3D face mesh. This face mesh is normalized and converted into 57 *blendshapes* (representing 52 facial expressions) ranging in value from 0 (neutral position) to 1 (pronounced expression), as well as five head position parameters (see **Figure 2**). Blendshapes were recorded with 10 fps.

From the ADOS videos of our nine participants, 158 video segments with a length of 5 seconds were selected by one of the authors, including smiling and neutral expressions. In these segments, the participants’ faces and facial features were clearly visible, i.e. faces were not covered by hands or objects, with frontal to semi-profile head rotation. The video segments were randomly assembled into one video file, which resulted in a continuous sequence of scenes.

Five human raters – who had no insight into the study design – were instructed to indicate whether a smile event (1) or a non-smile event (0) occurred in each segment. In the written instructions for the raters, we explained that every smile should be reported, regardless of the social function (e.g. embarrassed, socially desirable, inappropriate, joyful smile). Inter-rater agreement was very high, as indicated by a Fleiss’ Kappa of κ = 0.9101 (95% CI [0.8620, 0.9582]; p < 0.001). In the case of disagreement (n = 16), the majority vote applied. Human rating was set as the true class label, resulting in a binary coding for every of the 158 cases (0 = non-smile event, 1 = smile event).

### Threshold optimization for machine classification

The data were processed using Matlab (Version R2023a, MathWorks) (14). A random selection process was employed to allocate 50% of the smile and non-smile instances to the training data set and 50% to the test data set. This ensured that the participants and the smile/non-smile events in the two subsets were distributed as evenly as possible.

**Figure 3:**
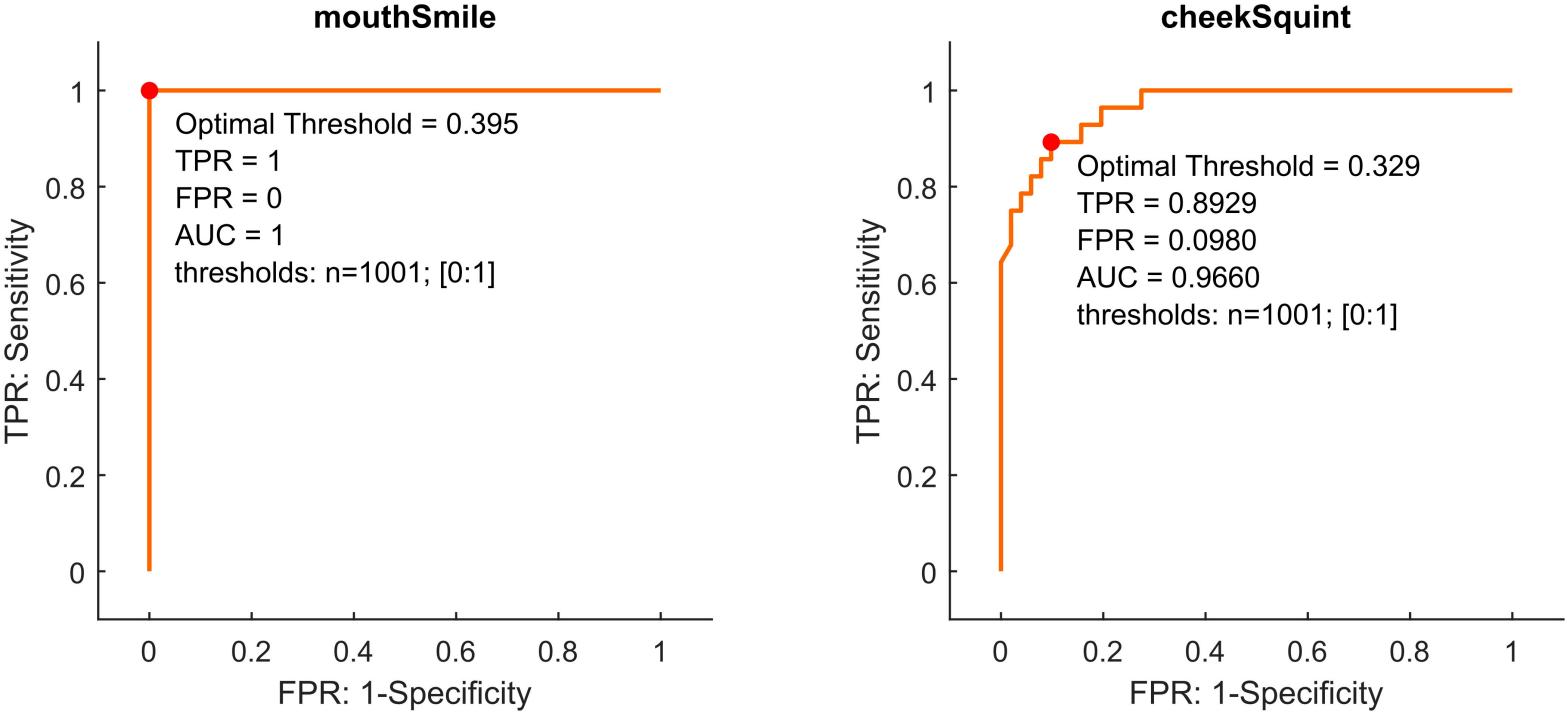
Receiver Operating Characteristics (ROC) of the classifiers *mouthSmile* and *cheekSquint*. The y-axis shows the true-positive rate (TPR) and the x-axis shows the false-positive rate (FPR) for each threshold. The area under the curve (AUC) of the ROC value of *mouthSmile* is equal to one and is therefore an excellent classifier for our training data.

Studies on smile recognition in healthy individuals found that AU6 (cheek raiser) and AU12 (lip corner puller) are most strongly associated with the expression of joy (15, 16). In the further analysis, we thus focused on four blendshapes (*cheekSquintLeft*, *cheekSquintRight*, *mouthSmileLeft* and *mouthSmileRight*) representing bilateral AU6 and AU12. The blendshapes of the left and right halves of the face were linked by a logical ‘or’ operation to compute classifiers that are more robust to changes in head position. For instance, the *mouthSmile* classifier was true, if either *mouthSmileLeft* ‘or’ *mouthSmileRight* exceeded the threshold. The same operation was applied for the *cheekSquint* classifier. For the 79 training cases, the receiver operating characteristics (ROC) were calculated for each classifier (see **Figure 3**). The human rater consensus was used as the true class label (smile/non-smile event). For ROCs, threshold values from 0 to 1 were employed, with an increment of +0.001 per step. The optimal threshold was identified through the application of Youden’s J statistic. The area under the curve (AUC) was calculated using trapezoidal numerical integration.

### Evaluation of the machine classification

The classifiers determined from the training data set were applied to the test data set. Sensitivity and specificity of the FER were calculated for each classifier. Cohen’s Kappa was used to assess the degree of agreement between the machine classification and the human rating.

## Results

### Receiver operating characteristics

ROCs (see **Figure 3**) show that a classification based on *mouthSmile* yielded a perfect classifier for the training data (AUC = 1). At the optimal threshold of 0.395 a true positive rate (TPR) of 1 was achieved, while the false positive rate (FPR) remained at 0. Classification based on *cheekSquint* demonstrated the capacity to differentiate between smile and non-smile events, but with lower accuracy (AUC = 0.966, TPR = 0.892, FPR = 0.098).

### Comparison of machine and human classification

The *mouthSmile* classifier (= either *mouthSmileLeft* or *mouthSmileRight* above threshold value) with the optimal threshold of 0.395 was employed to evaluate the test data set (n=79).

This resulted in a sensitivity of 0.964 and a specificity of 0.961, based on a confusion matrix with 49 true negatives, 27 true positives, 2 false positives, and 1 false negative. The degree of agreement between the machine classification and the human rating was assessed with Cohen’s Kappa, resulting in a value of κ = 0.9177 (95% CI [0.8263, 1.0090]; p < 0.001), indicating a very high level of agreement.

## Discussion

The key findings of the study are the following: [i] Smile events can be identified automatically with high sensitivity/specificity in selected sequences from ADOS videos, comparable with human ratings. [ii] Blendshapes representing the mouth corner puller are particularly robust to identify smile events. These results confirm the above hypotheses.

The aim of this pilot study was to demonstrate that the identification of smile events in an ASD diagnostics utilization population work reliable with automated FER.

Applied in a naturalistic setting, FER is challenging because the recording conditions are constantly changing: some children show a strong restlessness of movement, faces are occasionally covered by objects, hair or hands, the distance between camera and child is inconstant, and finally facial expressions are more subtle and spontaneous.

Thus, in this first step, an experimental setup was chosen that reduced interfering variables on the measurement. Only verbalized children with age > 6 years were included; video sequences were selected showing the probands faces completely and at an angle favourable for data acquisition. Moreover, unambiguous facial expressions were chosen (smile event, non-smile event). The results are therefore not generalizable and more extensive data collection is needed.

There are several studies that have examined automated recognition of basic emotions in healthy, predominantly adult subjects (15, 16). The facial expression of joy was recognized most reliably compared to other basic emotions. An extensive analysis of different video databases revealed that technical characteristics of each database (e.g. size of the visual field, extent of head rotation and movement) had a significant impact on recognition accuracy (15). Posed, prototypical expressions were recognized more reliably than spontaneous expressions (15). A study with 30 participants reported predictive accuracy of automatic identification of spontaneous smile events (based on AU6 and AU12) with a specificity of 82.9% and a sensitivity of 89.7% (16). Misclassified smiles were mostly related to covering the mouth and due to yawning. A study examining the difference between spontaneous and posed smile events reported differences between minors and adults (17).

Compared to the aforementioned studies, the FER in our study recognized smile events with a higher specificity and sensitivity. This is probably due to the more restrictive experimental conditions in our study. We investigated the identification of the facial expression of joy (smile event) in contrast to a neutral facial expression (non-smile event). Since the facial expressions of individual basic emotions overlap to some extent, studies that include videos of all basic emotions might probably achieve poorer results. The use of Apple ARKit, which allows to measure facial expressions with particular precision, might as well have made a secondary contribution to the positive key values.

In the present study, video snippets displaying a prototypical facial expression were analysed. An alternative approach could be to analyse the complete ADOS video datasets after prior annotation of the smile events by human raters. A key benefit of the proposed approach is that the technical feasibility of identifying smile events by FER can be readily substantiated. Furthermore, the determination of threshold values for the FER is more straightforward in a favourable recording position. A disadvantage of the comparison of the approaches is the poor generalisability of the results to the full ADOS situation and the lack of evidence for the reliability of the FER under unfavourable recording conditions.

In some studies, FER was applied to patients with ASD. For instance, Bangerter et al. found reduced mimic response behaviour (AU12, AU6) to an amusing video task in individuals with ASD compared to healthy controls (18). The study used software based on Computer Expression Recognition Toolbox (CERT) which, according to the authors, achieved an accuracy of 90.1% for a database of posed facial expressions and almost 80% for a dataset of spontaneous facial expressions. Ahn et al. used a first-person video approach by means of data glasses worn by parents and examiners to investigate smile events of 61 infants with ASD during an ADOS session (19). These data were also analyzed using the CERT-toolbox. Children whose gaze at their parents included more smiles received lower social affect severity scores (19). In a multicentre study, Perochon et al. investigated whether 49 children with ASD out of N=475 infants can be correctly classified using the app SenseToKnow (4). In addition to many other parameters, the complexity of mouth movement, measured with automated FER, was included in the assessment. The classification algorithm, combining multiple digital phenotypes based on machine learning, showed high diagnostic accuracy (area under the receiver operating characteristic curve = 0.90, sensitivity = 87.8%, specificity = 80.8%). These encouraging results illustrate the high potential of FER for ASD diagnostics.

This proof-of-principle study has shown that iOS-based automated recognition of smile-events in sequences from naturalistic ADOS-2 videos is successful in principle. Further investigations are required to verify whether identification of smile-events is successful in ADOS-segments under difficult conditions (e.g. profile recordings, low resolution of the recordings, partial facial masking). Moreover, it will be investigated which contribution the presented FER may provide for the identification of children at risk of ASD. The identification and quantification of smile events in non-verbal communication (e.g. as reciprocal smiles) might be of particular importance. There are many conceivable areas of application for automated facial expression analysis in ASD diagnostics, for example as a supplement to the standard questionnaire-based autism screening or as an objective assistance system for analyzing ADOS examinations. The results described are preliminary and only partially utilize the possibilities of FER. In particular, a dedicated analysis of pooled data sets and AI-based identification of discrepant features of social communication open up new, promising approaches.

## Strengths and limitations

This study demonstrated that automated identification of smile events in selected video sequences of a naturalistic ADOS-2 setting is both possible and reliable. This was the aim of this proof-of-principle study.

This pilot study is subject to various limitations: 1) Only video sequences with clear, prototypical smile events in a predominantly frontal view were selected, 2) narrow inclusion criteria (verbalization, age range, no cognitive impairment) were applied, 3) participants studied showed moderate ASD symptoms and did not meet ASD diagnostic criteria. Thus, results cannot be generalized all ADOS-2 participants or –ADOS-2 settings. Nevertheless, the results point to interesting opportunities and research desiderata regarding the use of advanced FER technology in the clinical setting.

## Data Availability

The raw data supporting the conclusions of this article will be made available by the authors, without undue reservation.
